# ONE-LEGGED STANCE BALANCE OF OLDER ADULTS WITH AND WITHOUT FALLS

**DOI:** 10.1093/geroni/igz038.1754

**Published:** 2019-11-08

**Authors:** Edgar R Vieira, Márcio Oliveira, Andre Gil, Karen Fernandes, Denilson Teixeira, Cesar F Amorim, Rubens da Silva

**Affiliations:** 1 Department of Physical Therapy, Florida International University, Miami, Florida, United States; 2 Clinical Master’s Program in Physical Exercise in Health Promotion, Pitagoras Unopar, Londrina, Parana, Brazil; 3 Department of Physical Therapy, Pitagoras Unopar, Londrina, Parana, Brazil; 4 Graduate Programs in Rehabilitation Sciences UEL/UNOPAR, Londrina, Parana, Brazil; 5 Department of Physical Education, Universidade Estadual de Londrina, Londrina, Parana, Brazil; 6 Departement des Sciences de la Sante, Universite du Québec à Chicoutimi (UQAC), Saguenay, Quebec, Canada

## Abstract

Balance impairment is a common problem among older adults. Poor balance in older adults is often associated with mobility impairments, activity limitations and fear of falling in older adults. Thus, balance assessment is useful for early detection of postural control deficits to prevent mobility impairments and falls in older adults. The aim of this study was to assess if balance measures based in center of pressure (COP) parameters during one-legged stance could differentiate between older adults with and without falls in the past 12 months. One-hundred and seventy older adults (50 fallers and 120 non-fallers, age range: 63-72 years) performed three 30s one-legged stance trials with eyes open on a force platform with 30s of rest between each trial. The following variables were evaluated: COP 95% elliptical area, COP velocity in the anterior-posterior and medio-lateral directions, and test duration (how long the participant was able to stay in one-legged stance, up to 30s). Fallers had poorer balance than non-fallers (P ≤0.004). The COP parameters presented an area under the curve between 0.65-0.72, with sensitivity varying from 66 to 78% and specificity from 54 to 68%. There were no significant differences between fallers and non-fallers on test duration (17 vs. 18s, respectively). The findings showed that the fallers had similar duration time, but poorer balance than the non-fallers during one-legged stance. The COP parameters were able to differentiate the balance between fallers and non-fallers with acceptable area under curve, sensitivity and specificity.

